# Web-Based Delivery of a Family-Based Dating Abuse Prevention Program for Adolescents Exposed to Interparental Violence: Feasibility and Acceptability Study

**DOI:** 10.2196/49718

**Published:** 2023-12-01

**Authors:** H Luz McNaughton Reyes, Eliana G Armora Langoni, Laurel Sharpless, Kathryn E Moracco, Quetzabel Benavides, Vangie A Foshee

**Affiliations:** 1 Department of Health Behavior Gillings School of Global Public Health University of North Carolina at Chapel Hill Chapel Hill, NC United States

**Keywords:** dating violence, adolescents, family-based prevention, web-based delivery, feasibility and acceptability, mobile phone

## Abstract

**Background:**

Numerous studies have demonstrated that exposure to caregiver intimate partner violence (IPV) can have cascading negative impacts on children that elevate the risk of involvement in dating abuse. This cascade may be prevented by programs that support the development of healthy relationships in children exposed to IPV. This paper describes the results of a study of the web-based adaptation of an evidence-based dating abuse prevention program for IPV-exposed youth and their maternal caregivers. Core information and activities from an evidence-based program, Moms and Teens for Safe Dates, were adapted to create the web-based program (e-MTSD), which comprises 1 module for mothers only and 5 modules for mother-adolescent dyads to complete together.

**Objective:**

The primary objective of this study was to evaluate the feasibility and acceptability of the e-MTSD program and the associated research processes. We also examined the practicability of randomizing mothers to receive SMS text message reminders and an action planning worksheet, which were intended to support engagement in the program.

**Methods:**

Mothers were recruited through community organizations and social media advertising and were eligible to participate if they had at least one adolescent aged 12 to 16 years of any gender identity who was willing to participate in the program with them, had experienced IPV after their adolescent was born, and were not currently living with an abusive partner. All mothers were asked to complete the program with their adolescent over a 6- to 8-week period. Participants were randomized to receive SMS text message reminders, action planning, or both using a 2×2 factorial design. Research feasibility was assessed by tracking recruitment, randomization, enrollment, and attrition rates. Program feasibility was assessed by tracking program uptake, completion, duration, and technical problems, and acceptability was assessed using web-based surveys.

**Results:**

Over a 6-month recruitment period, 101 eligible mother-adolescent dyads were enrolled in the study and were eligible for follow-up. The median age of the adolescent participants was 14 years; 57.4% (58/101) identified as female, 32.7% (33/101) identified as male, and 9.9% (10/101) identified as gender diverse. All but one mother accessed the program website at least once; 87.1% (88/101) completed at least one mother-adolescent program module, and 74.3% (75/101) completed all 6 program modules. Both mothers and adolescents found the program to be highly acceptable; across all program modules, over 90% of mothers and over 80% of adolescents reported that the modules kept their attention, were enjoyable, were easy to do, and provided useful information.

**Conclusions:**

Findings suggest the feasibility of web-based delivery and evaluation of the e-MTSD program. Furthermore, average ratings of program acceptability were high. Future research is needed to assess program efficacy and identify the predictors and outcomes of program engagement.

## Introduction

### Background

Approximately 1 in 4 children in the United States are exposed to intimate partner violence (IPV) between their parents or other caregivers before the age of 18 years with an estimated 15.5 million children exposed each year [1,2]. Numerous studies have demonstrated that exposure to caregiver IPV has cascading negative impacts on cognitive, emotional, and social development that, in turn, increases children’s risk of becoming involved in abusive romantic relationships during adolescence and adulthood. This cascade, sometimes referred to as the i*ntergenerational transmission of IPV* [2], may be prevented by programs that promote resilience and family healing and foster healthy development in children exposed to IPV. However, to date, few such programs have been designed to address the specific needs of IPV-exposed youth or to evaluate for impacts on violence outcomes [3,4]. Furthermore, few programs have been developed that focus on engaging caregivers as prevention agents [5]. One exception is Moms and Teens for Safe Dates (MTSD), a family-based dating abuse prevention program developed for youth who have been exposed to IPV and their maternal caregivers (henceforth referred to as “mothers” and inclusive of nonbinary people, gender expansive people, and transgender women who identify as moms or maternal caregivers) who experienced the abuse [6].

The MTSD program comprises a series of booklets with activities for mothers and their 12- to 16-year-old adolescents to complete together that are designed to foster a family environment that protects against adolescent dating violence. A randomized controlled trial (RCT) of the MTSD program conducted in 2012 found that the program was effective in increasing family cohesion and preventing dating abuse among youth with high, but not low, levels of IPV exposure [7]. The results of this trial are promising, particularly given that the MTSD program is relatively low cost, in that it is self-administered versus staff administered, and is convenient to complete, in that families can work to complete the booklets at times and in locations of their choice. However, there are several characteristics of the program that may prevent it from being widely disseminated and implemented. The costs of printing and disseminating booklets may be prohibitive to organizations serving IPV-exposed mothers, which are typically low resourced. Furthermore, there are no built-in cues or reminders to complete the program, which may lead to low engagement in real-world settings, and the relatively high reading burden may limit reach to lower literacy participants.

To address these dissemination and implementation barriers, we developed a web-based MTSD (e-MTSD) program [8]. In addition to eliminating booklet printing costs, web-based delivery has the potential to (1) lower reading burden via audiovisual presentation of information and activities, (2) allow low-cost built-in delivery of tailored reminders to engage with the program based on website use monitoring, (3) increase convenience in that participants can log in to the intervention via an internet-enabled device (eg, smartphone or tablet) at any time point, and (4) increase program appeal to digitally oriented adolescents. Thus, if found to be feasible and acceptable to participants, as well as efficacious in preventing dating abuse involvement, e-MTSD has high potential for scale-up and public health impact.

### Objectives

The overarching goal of this study was to determine whether the e-MTSD program was appropriate for further testing. This study has 3 main objectives. First, we examined the feasibility of the research process used to evaluate the program. Limited research has sought to evaluate programs for caregiver-adolescent dyads using web-based recruitment, assessment, and program-delivery methods. Therefore, a better understanding of the practicality of the proposed research processes may inform future evaluation efforts. Second, we assessed the acceptability and feasibility of the e-MTSD program. Acceptability was defined as participants’ “emotional and cognitive responses to the program” [9]. Feasibility was conceptualized as the extent to which participants were able to engage with and complete the web-based intervention. Third, we explored the acceptability and feasibility of delivering two distinct implementation supports: (1) SMS text message reminders and (2) completion of an “action planning” worksheet that we hypothesized might increase engagement with the program. The feasibility of delivering different combinations of these implementation supports was explored based on research that suggests that digital triggers such as SMS text messages [10] and the development of an “action plan” that details when, where, and how a participant proposes to complete program activities [11] may work to increase engagement.

## Methods

### Participants

Mother-adolescent dyads were recruited for the study via social media posting and information dissemination through community agencies (eg, domestic violence organizations) and educational institutions (eg, community colleges) that work with or provide services to mothers and IPV survivors. Mothers residing in the United States were eligible to participate if they (1) had at least one 12- to 16-year-old child of any gender identity who lived with them at least part of the time; (2) had experienced IPV at some point in their lives after at least one of their 12- to 16-year-old children was born; (3) were not currently living with an abusive partner; (4) were able to read and speak English; (5) had access to an internet-enabled device; and (6) were able to receive SMS text messages. Mothers who had more than one age-eligible child were asked to select 1 child to participate in the study together with them.

### Procedures

#### Overview

Recruitment materials were directed at mothers of children aged 12 to 16 years. Recruitment messages were positive and gain framed, focused on the benefits of the program for “helping mothers prepare teenagers to engage in healthy relationships.” Messages explicitly stated that the program was developed for mothers or maternal caregivers who had experienced domestic violence in the past and had a 12- to 16-year-old adolescent. We used a multimethod recruitment plan that included a range of outlets (email blasts, listservs, social media posts and advertisements, flyers, and brochures). Study advertisements directed individuals interested in participating in a link where they could complete a web-based prescreening questionnaire and provide contact information and permission to be contacted. In addition, materials included the study phone number, email, and web address for a recruitment website that included more information about the study procedures and directed interested individuals to the eligibility screener link. The research staff reviewed prescreening questionnaires and attempted to schedule a study orientation via telephone with mothers who were screened as potentially eligible and provided consent to be contacted. Mothers who were contacted by phone were administered a standard set of questions to confirm eligibility, and those who were eligible were provided with study details and asked to confirm their interest in enrollment. All study communications (ie, phone calls, emails, and texts) were conducted with potentially eligible or enrolled mothers. The research staff did not collect contact information or directly communicate with potentially eligible or enrolled adolescents. Mothers who indicated that they were interested in enrolling were sent an electronic consent form to complete as well as an electronic assent for their adolescent to review and complete. Once the consent and assent forms were completed, mothers were sent a link to a baseline survey to complete as well as a link to a separate baseline survey for their adolescent to complete. Eligible mother-adolescent dyads who completed the baseline surveys were enrolled in the study.

#### e-MTSD Program Structure and Exposure Window

Mothers who logged into the e-MTSD program were directed to view a brief onboarding video explaining the program and then complete the Getting Started module, which is designed to prepare and motivate mothers to complete the program with their adolescent. At the completion of the Getting Started module, mothers were instructed to complete the remaining 5 program modules together with their adolescent. These modules included information and interactive activities designed to increase mother-adolescent positive communication about healthy and unhealthy relationships and reduce the adolescent’s risk of experiencing dating violence. All mothers who enrolled in the study were asked to complete all 6 modules of the e-MTSD program together with their adolescent over a 6-week period, with a 2-week grace period allowed for families who were unable to comply with this schedule (56-d maximum program exposure interval). One week before losing access to the program (day 49), all participants were sent an email and SMS text message reminder that their access to the program was going to expire. For additional details on the e-MTSD program development process, content, and structure, see the study protocol paper [8].

### Design

#### Overview

All enrolled mothers, regardless of group assignment, were provided information on how to access and log in to the program via email and were sent a welcome text directly from the web-based program. In addition, mother-adolescent dyads were randomized to 1 of 4 “adherence support” groups (conditions): text reminders only (TR), action planning only (AP), text reminders plus action planning (TRAP), or low adherence support (LA). We randomly assigned participants to conditions to determine whether it was feasible to deliver different combinations of support to participant dyads in anticipation of potentially testing the main and interactive effects of different supports on engagement outcomes (eg, amount of program use) in a future RCT.

#### Adherence Supports Common to All Conditions

All mother participants who had been sent onboarding information and who did not log in to the program and complete Getting Started (the mothers-only module) received up to 3 reminders via text 3, 7, and 10 days after enrollment. These reminders prompted mothers to log in and offered technical support if needed. Mothers could text back in response to these reminders to solicit technical support by logging in to or navigating the program. All mother participants who had not completed the program 49 days after enrollment were sent a notification that their access to the program would end in 7 days and were offered technical support if needed.

#### Adherence Supports That Differed Between Conditions

Mother participants in the TR and TRAP conditions received up to 8 tailored messages at fixed intervals. Messages 1, 2, and 3 were sent 3, 7, and 10 days after the onboarding information and welcome text were sent. Messages 4 through 8 were sent every 7 days thereafter until the program was finished. Reminders were tailored based on whether the dyad was on track with respect to the recommended completion rate of 1 module per week. Those who were on track or ahead of schedule were sent a congratulatory message. Those who were behind schedule received messages that aimed to motivate engagement (eg, “this fun and convenient program has been shown to benefit moms and teens*”*) and offer technical support. Mothers assigned to the AP and TRAP conditions were provided with a modified version of the onboarding information that asked them to complete a brief electronic form before logging into the program that prompted them to make an “action plan” for where and when they would complete the program and identify how they would overcome barriers to engaging with the program.

### Ethical Considerations

This study was approved by the University of North Carolina at Chapel Hill Institutional Review Board (study # 21-2380). Informed consent was obtained from maternal caregivers, and assent was obtained from adolescent participants. All study communications were with maternal caregivers and not directly with adolescents. Mothers were asked to ensure their adolescent’s privacy when completing the web-based surveys. All data were collected and stored using secure servers, and data were deidentified before analysis. Participants were compensated with a US $30 gift card upon completion of baseline, program completion, and completion of follow-up surveys (US $90 total/participant).

### Measures

#### Overview

Mother and adolescent participants completed brief module acceptability surveys, which were embedded in the e-MTSD program at the end of each program module (n=6 surveys). In addition, participants completed web-based surveys at baseline and at two additional assessment points: (1) immediately after program completion or, if the program was not completed during the exposure window, 56 days after enrollment (postprogram survey) and (2) 90 days after enrollment (follow-up survey). Questions within the postprogram survey were tailored depending on (1) how much of the program had been completed and (2) adherence support group assignment. Specifically, participant dyads who had not completed the program were asked about barriers that may have prevented them from engaging with the program. Mother participants assigned to the text reminder and action planning condition were asked about the helpfulness of these adherence supports.

e-MTSD is designed for participants to move sequentially in a forward progression through 6 modules with the ability to return to completed content. To track program use, we assigned a number to each page in the program sequence (n=107) and classified each page as providing an introductory video, an interactive activity, information only, or a module completion survey. Web paradata on program use were collected for all participants who logged into the program (n=100) and included three variables: (1) the participant’s identification number; (2) the visited URL, which corresponded to a page number in the program sequence; and (3) the date and time the page was accessed. There were 13,170 data points in the use database for all the participants. The mean by participant was 131.7 (SD 94.0; range 1-649). There were 4473 duplicate page views (due to a participant logging out and in again, browser refresh, or return view). We used this database to create indicators of program uptake, use, and duration (described in subsequent sections).

#### Participant Characteristics

Mother participants reported their relationship with the adolescent participating in the study with them, age, self-identified race and Hispanic ethnicity, highest level of education, whether they were currently living with a partner, and whether they had ever petitioned for a domestic violence protective order. To assess family financial stress, mother participants were asked, “Thinking about the next month, how worried are you that you and your family will have difficulty with having enough to eat?” [12]. Responses of “very” and “somewhat” worried were coded as 1 and responses of “not too” and “not at all” worried were coded as 0. To assess exposure to different types of violence, we asked mothers to report whether, at any time in their life, a partner had (1) pushed, slapped, hit, punched, kicked, choked, or beat them up; (2) repeatedly sworn at, insulted, put down, or humiliated them; and (3) forced them to do sexual things they did not want to. For items 1 and 2, we further asked mothers whether, at any time in their life, the adolescent that was participating in the study with them had ever seen or heard this happen to them (mother-reported adolescent IPV exposure).

Adolescent participants reported their age, self-identified race, Hispanic ethnicity, gender identity [13], sexual identity [13], and dating status, which was assessed by asking adolescent participants whether they had ever “dated, gone out, hooked up, or been in a romantic relationship with someone.” Dating violence was assessed using items that measured lifetime experiences of psychological (n=5 items; eg, “they insulted my looks clothes, or appearance, and it made me feel bad, embarrassed, or insecure”), physical (n=3 items; eg, “they twisted my arm, slapped, pushed, shoved, or shook me”), and sexual dating violence victimization (n=5 items; eg, “they pressured me to do something sexual”). New binary variables were then created for each type of violence (ie, psychological, physical, or sexual) such that a score of “1” indicated that the participant had endorsed at least 1 item assessing that type of violence and “0” otherwise. Parallel items and scoring procedures were used to assess the perpetration of dating violence. See Tables S1 and S2 in Multimedia Appendix 1 [7,12,13,14] for a list of items, original response codes, and item sources for data reported on participant characteristics.

#### Feasibility Indicators

##### Recruitment, Enrollment, Randomization, and Retention Rates

We calculated the recruitment rate as the average number of potentially eligible participants (per initial screening) recruited per month. The enrollment rate was calculated as the percentage of participant dyads confirmed eligible (via telephone screen) who enrolled in the study (ie, they completed consent and assent forms as well as the baseline survey). The randomization rate was calculated as the proportion of enrolled participants who were correctly randomized to an adherence support condition. Retention rate was calculated as the percentage of participants eligible for follow-up who completed the postprogram and follow-up surveys.

##### Adherence Support Delivery

We assessed the delivery of SMS text messages via the program completion survey by asking participants in the SMS text message support conditions whether they had or had not received SMS text message reminders to complete the program. Delivery of action planning was assessed by calculating the proportion of mother participants assigned to the AP and TRAP conditions who completed the action planning form.

##### Data Collection

For the baseline survey, which was the longest survey used in this study, we assessed the following: (1) the percentage of missing data for each item in the survey, (2) the percentage of missing items for each respondent, and (3) the time taken to complete the survey.

##### Program Uptake

We defined program uptake using two indicators: (1) the proportion of enrolled participants who logged in to the program at least once and (2) the proportion of enrolled participants who completed module 1, indicating that the participant mother had started the program with their adolescent.

##### Program Use

Program use was operationalized as the percentage of modules completed, and program completion was operationalized as whether a participant had completed all program modules. A module was coded as completed if the participant was recorded as having visited all the unique pages in the module.

##### Program Duration

Following previous research, we operationalized duration as the overall time spent to complete a module and the full program [15]. e-MTSD did not “time out” or include an end time stamp when participants moved to the next page. Rather, the time stamp represented when the user first accessed a page, and the next stamp entry denoted the next page accessed. The time spent on the page was calculated as the difference between these access points. Some pauses in the time stamped data were greater than the presumed time that would be spent on a page (eg, several hours, days, or weeks), signifying that the user was no longer engaged with the program. Following a series of steps outlined by Breitenstein et al [15], we dealt with this issue by imputing the data as follows. First, we assessed the distribution of page visit times by page type. We found that information pages were viewed for a shorter time (median 14 seconds) than video (median 26 seconds), activity (median 39 seconds), and survey (median 55 seconds) pages. Distributions had many outliers, with outliers most typical for pages at the start and end of each module, likely indicating a break in engagement. We calculated an upper “fence” for each page type as 1.5 times the IQR. The introductory video pages had the largest upper fence (464 seconds) and the information pages had the smallest (94 seconds). We chose to use the 464-second (approximately 8 min) cutoff, which would ensure that most durations not imputed were likely reasonable estimates of how long a family would maximally remain engaged on a page. Therefore, all page visit durations greater than 464 seconds were replaced with the median value for their page type. Module duration was calculated for each participant who completed a module by summing the total time across all pages within a module (after imputation of page times). Program duration was similarly calculated for all those who completed the program by summing page visit times across all pages visited.

##### Technical Problems

Technical problems were assessed in each module completion survey via a question that asked whether participants had experienced technical problems using or accessing the website and via an open-ended follow-up question asking what technical problems occurred.

#### Acceptability Indicators

##### Program and Module Acceptability

Primary indicators of acceptability cover each of the domains specified in the Theoretical Framework of Acceptability, including affective attitude, burden, perceived effectiveness, ethicality, intervention coherence, opportunity costs, and self-efficacy [16]. Response options for each indicator (eg, I enjoyed doing this module [program]) ranged from strongly disagree (score=0) to strongly agree (score=3). Owing to skewed distributions of responses, we recoded the indicators such that responses of agree or strongly agree were coded as “1” and responses of disagree and strongly disagree were coded as “0.” We also included 1 general question that asked whether there were “any parts of the module that you disliked doing” and an open-ended follow-up question about what they did not like. Secondary indicators of acceptability included participant responses on the postprogram survey to 10 indicators that asked participants whether the program had achieved specific impacts (eg, “the program taught me how to recognize dating abuse”). The response options were “yes” or “no.” These indicators were only included in the postprogram survey if the mother and adolescent had completed at least one program module together.

##### Adherence Support Acceptability

In the postprogram survey for mothers, participants assigned to the TR and TRAP conditions were asked how helpful the SMS text reminders were for them to do the program with their adolescent. We coded responses such as “very helpful” and “somewhat helpful” as 1 and responses such as “not at all helpful” and “not very helpful” as 0. In addition, participants were asked if they felt that the number of SMS text messages received was “too many,” “just the right number,” or “too few.” Participants who were assigned to the AP and TRAP conditions were asked how helpful or not helpful it was to make the action plan before completing the program. We coded responses such as “very helpful” and “somewhat helpful” as 1 and responses such as “not at all helpful” and “not very helpful” as 0.

## Results

### Participant Characteristics

The characteristics of mother (Table 1) and adolescent (Table 2) participants are presented for 101 mother-adolescent dyads who enrolled in the study and were eligible for follow-up.

Nearly all mother participants (98/101, 97%) reported that they were the participating adolescents’ biological or adoptive mother. Approximately 9.9% (10/101) reported that their highest level of education was high school or less and 24.8% (25/101) reported being somewhat or very worried about their family having enough to eat in the next month. Most mother participants (70/101, 69.3%) reported that they had petitioned for a domestic violence protective order, 94.1% (95/101) reported having experienced psychological IPV, 84.2% (85/101) reported that they had experienced physical IPV, and 65.3% (66/101) reported that they had experienced sexual IPV.

The median age of the adolescent participants was 14 years, and age was approximately evenly distributed across the age range in the sample (12-16 years). Approximately 17.8% (18/101) of adolescent participants reported their race and ethnicity as non-Hispanic Black or African American, 14.9% (15/101) reported as Hispanic or Latino 46.5% (47/101) reported as non-Hispanic White; and 20.8% (21/101) reported as non-Hispanic multiracial or “other” race. Approximately one-third (33/101, 32.7%) of the adolescents identified as male; 57.4% (58/101) identified as female; and 9.9% (10/101) identified as “other” gender identity. Most (72/101, 71.3%) adolescents self-reported their sexual identity as “straight or heterosexual”; 12.9% (13/101) reported their sexual identity as bisexual; 4% (4/101) identified as gay or lesbian; and 11.9% (12/101) identified as “other.” See the note in Table 2 for a detailed breakdown of the “other” categories for gender identity, race and ethnicity, and sexual identity. According to mother participant reports, approximately 54.5% (55/101 of adolescents had ever seen or heard their mother experience physical IPV and 79.2% (80/101) had ever seen or heard their mother experience psychological violence. In terms of dating and adolescent dating violence exposure, 70.3% (71/101) of adolescents reported having ever dated, gone out, hooked up, or been in a romantic relationship with someone. Nearly half (49/101, 48.5%) reported having experienced psychological victimization in a dating relationship, 15.8% (16/101) reported physical victimization, and 24.8% (25/101) reported sexual victimization. The rates of perpetration were lower, with 20.8% (21/101 of adolescents reporting psychological perpetration, 10.9% (11/101) reporting physical perpetration, and 6.9% (7/101) reporting sexual perpetration.

**Table 1:** Characteristics of participant mothers (n=101).

Characteristics			Values
**Relationship to the teenager, n (%)**			
	Biological mother, adoptive mother, or stepmother	99 (98)	
	Other (grandmother or foster mother)	2 (2)	
Age (years), median (range)			40 (27-58)
**Race and ethnicity, n (%)**			
	Black or African American and non-Hispanic	17 (16.8)	
	Hispanic or Latina	16 (15.8)	
	White non-Hispanic	57 (56.4)	
	Other or non-Hispanic^a^	11 (10.9)	
**Educational attainment, n (%)**			
	High school graduate or less	10 (9.9)	
	Some college or no degree	24 (23.8)	
	College degree	67 (66.3)	
Currently living with a partner, n (%)			40 (39.6)
Somewhat or very worried about family having enough to eat in the next month, n (%)			25 (24.8)
Ever filed for a domestic violence protective order, n (%)			70 (69.3)
**Violence exposure, n (%)**			
	Ever repeatedly sworn at, insulted, or humiliated by a partner	95 (94.1)	
	Ever pushed, slapped, hit, punched, kicked, choked, or beat up by a partner	85 (84.2)	
	Ever forced to do sexual things they did not want to by a partner	66 (65.3)	

^a^The other race-ethnicity category included mothers who identified as American Indian or Alaska Native (n=4), Asian (n=3), Native Hawaiian or Pacific Islander (n=1), multiracial (n=6), and other (n=2).

**Table 2:** Characteristics of participant adolescents (n=101).

Characteristics				Values
Age (years), median (range)				14 (12-16)
**Gender identity, n (%)**				
	Female		58 (57.4)	
	Male		33 (32.7)	
	Other gender identity^a^		10 (9.9)	
**Race and ethnicity, n (%)**				
	Black or African American and non-Hispanic		18 (17.8)	
	Hispanic or Latina		15 (14.9)	
	White or non-Hispanic		47 (46.5)	
	Multiracial or non-Hispanic		10 (9.9)	
	Other or non-Hispanic^b^		11 (10.9)	
**Sexual identity, n (%)**				40 (39.6)
	Bisexual		13 (12.9)	
	Gay or lesbian		4 (4)	
	Straight or heterosexual		72 (71.3)	
	Other^c^		12 (11.9)	
**Adolescent IPV^d^ exposure**				
	Ever seen or heard mother get pushed, slapped, hit, punched, kicked, choked, or beat up by a partner^e^		55 (54.5)	
	Ever seen or heard mother be repeatedly sworn at, insulted, or humiliated by a partner^e^		80 (79.2)	
**Dating and dating violence exposure, n (%)**				
	Ever dated, gone out, hooked up, or been in a romantic relationship with someone		72 (71.3)	
	**Ever experienced ADV^f^ victimization**			
		Psychological	49 (48.5)	
		Physical	16 (15.8)	
		Sexual	25 (24.8)	
	**Ever perpetrated ADV**			
		Psychological	21 (20.8)	
		Physical	11 (10.9)	
		Sexual	7 (6.9)	

^a^The “other” gender identity includes adolescents who identified as transgender (n=2), gender nonconforming (n=2), nonbinary (n=5), and undisclosed (n=1).

^b^The “other” race and ethnicity category includes adolescents who identified as American Indian or Alaska Native (n=7); Asian (n=1); Native Hawaiian or Pacific Islander (n=1); and other (n=2).

^c^The “other” sexual identity category includes adolescents who identified as Pansexual (n=5); unknown (n=3); Asexual (n=2); and undisclosed (n=1).

^d^IPV: intimate partner violence.

^e^On the basis of a mother’s report.

^f^ADV: adolescent dating violence.

### Feasibility

#### Recruitment, Enrollment, Randomization, and Retention

**Figure 1:**
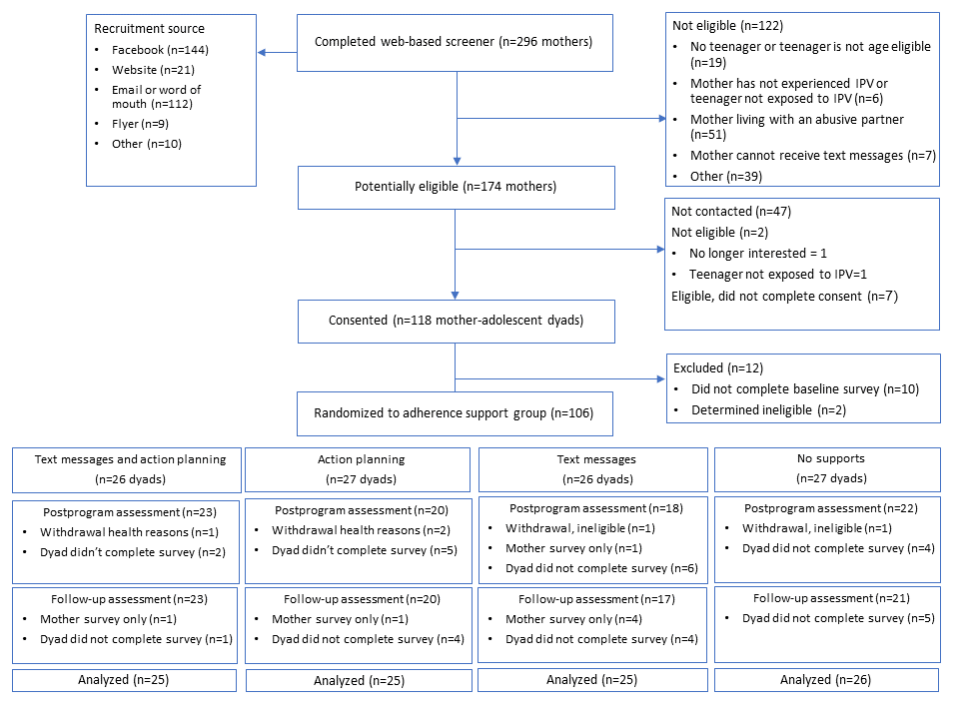
CONSORT (Consolidated Standards of Reporting Trials) flow diagram of the study. IPV: intimate partner violence.

Figure 1 provides a CONSORT (Consolidated Standards of Reporting Trials) flow diagram of the study progress. Recruitment began in mid-November 2021 and ended in May 2022 (approximately 6-month period). Over the recruitment period, 296 screeners were completed (approximately 49/month), of which 174 (58.8%) were found to be potentially eligible for participation, yielding a monthly recruitment rate of approximately 29 potentially eligible participants per month. Of the 174 potentially eligible participants, we called and confirmed the eligibility of 125 (71.8%) participants. A total of 106 mother-adolescent dyads completed consent or assent forms and baseline surveys, yielding an enrollment rate of 84.8% (106/125), or approximately 18 enrolled mother participants per month. All 106 enrolled participants were randomized as follows: 26 dyads were assigned to the TR and TRAP groups and 27 dyads were assigned to the AP and LA groups. After randomization, 3 participant dyads withdrew from the study due to health reasons and 2 were withdrawn after being found ineligible, leaving 101 dyads eligible to complete the postprogram and follow-up surveys (Figure 1). In total, 84 (83.2%) mothers and 83 (82.2%) adolescents completed the postprogram survey; 87 mothers and 81 adolescents completed the follow-up survey. The retention rates did not differ significantly by condition. We examined the recruitment sources for participants who enrolled in the study, excluding 2 participants who were later found ineligible (n=104). Nearly all participants indicated that they heard about the study through Facebook (35/104, 33.7%) or via email or word of mouth (51/104, 49%). There was no association between recruitment source (Facebook vs other) and mother participant demographic characteristics (reported in Table 1). A total of US $336 was spent on Facebook advertising, resulting in total expenditure of approximately US $10 per enrolled participant.

#### Data Collection

In the baseline surveys for mothers and adolescents, there were 45 and 90 items, respectively, for which data were missing for at least one respondent (not counting items with missing data due to valid skip patterns). Of these items, nearly all (>90%) were missing for only 1 or 2 respondents in both the mother and adolescent surveys. One item, which was the same item on both surveys, had more substantial missing data (n=12 missing adolescent and n=8 missing mother observations). In both surveys, this item involved the use of a “slider” to enter responses. Previous research has shown that sliders produce more incomplete data than clickable radio buttons in previous research (Funke 2016). At the participant level, nearly all (88% for both mothers and adolescents) of the 43 adolescents and 41 mothers had missing data on 1 or 2 items. One adolescent participant had more substantial missing data (25% of all survey items they were eligible to complete were missing). The baseline survey completion time was assessed for 46 mother and 38 adolescent participants; the median time to complete the survey was 22 minutes for mothers and 25 minutes for adolescents.

#### Program Uptake, Use, and Duration

Table 3 presents descriptive information on each e-MTSD program module, including the number of pages in each module by page type (activity or video, informational, or survey), the number and proportion of participants who completed the module, and time to completion. All but 1 participant accessed the site at least once (n=100), and 88% (88/100) of dyads completed module 1. Five (5%) participant dyads did not complete any module; 11 (11%) completed only 1 module (8 completed only Getting Started and 3 completed only module 1 owing to a program glitch that led them to skip Getting Started); 9 (9%) completed 2 modules (Getting Started and module 1); 1 (1%) completed 4 modules (Getting Started and modules 1-3); and 75 (75%) completed all modules. Program completion rates were highest for those in the TRAP condition (84/100, 84%), followed by the LA (73/100, 73%), AP (72/100, 72%), and TR (68/100, 68%) conditions; however, the association between condition assignment and program completion was not statistically significant (*χ*^2^=1.8, n=101; *P*=.61). Among those who completed the full program, the median time to program completion was 146 minutes (approximately 2.5 hours). Modules 1, 2, and 3 each took a median time of approximately 28 minutes to complete; the Getting Started module and modules 4 and 5 were shorter, with median completion times of 16, 17, and 18 minutes, respectively.

**Table 3:** Pages and page types, percent completed (n=101), and median time to completion by using the web-based Moms and Teens for Safe Dates program module^a^.

Module	Number of pages by page type, n (% of total pages in module)					Module completed, n (%)		Time to complete by module, median (IQR; range)
	Activity and video page	Informational only	Survey	Total				
Getting started	8 (44.4)	9 (50)	1 (5.6)	18 (100)	93 (92.1)		19 (10; 3-51)	
Module 1	14 (70)	5 (25)	1 (5)	20 (100)	88 (87.1)		28 (22; 7-75)	
Module 2	12 (66.7)	5 (27.8)	1 (5.6)	18 (100)	76 (75.2)		28 (20; 6-75)	
Module 3	12 (70.6)	4 (23.5)	1 (5.9)	17 (100)	76 (75.2)		27 (22; 5-121)	
Module 4	7 (46.7)	7 (46.7)	1 (6.7)	15 (100)	75 (74.3)		16 (14; 2-52)	
Module 5	15 (78.9)	3 (15.8)	1 (5.3)	19 (100)	75 (74.3)		17 (19; 4-58)	
Full program	58 (54.2)	33 (30.8)	6 (5.6)	107 (100)	75 (74.3)		146 (103; 37-370)	

^a^The program home page was included in the module 1 page count. Module completion is indicated by viewing all the pages in the module. Owing to a technical glitch, 3 participants who did not complete the Getting Started module completed module 1.

#### Technical Problems

The proportion of participants who reported experiencing technical problems with the program after each module (eg, problems with logging in and glitches in activities) differed across modules (range 3%-16%), with the greater proportion of participants reporting problems after completing modules with more activity pages (modules 1, 2, and 3). A review of open-ended responses to each module completion survey identified a set of specific issues (eg, after page refresh or logout participants made to start over) raised by multiple respondents that should be reviewed and addressed before subsequent evaluation of the program (Table S3 in Multimedia Appendix 1).

### Acceptability

#### e-MTSD Program Acceptability

Over 90% of the mother and adolescent respondents to the postprogram survey agreed with the indicators of program acceptability corresponding to the theoretical domains of affective attitude, effectiveness, ethicality, cohesiveness, self-efficacy, and opportunity costs (Table 4). More mothers (63/84, 75%) than adolescents (52/83, 63%) reported that they *disagreed* that the program was too long.

**Table 4:** Frequency and percentage of study participants endorsing indicators of web-based Moms and Teens for Safe Dates program acceptability^a^.

Program acceptability items	Theoretical domain	Mother (n=84), n (%)	Adolescent (n=83), n (%)
I enjoyed doing the program	Affective attitude	84 (100)	78 (94)
My teen enjoyed the program	Affective attitude	76 (95)^b^	N/A^c^
The program was easy to do	Burden	83 (99)	80 (96)
I understood what the program was trying to do	Cohesiveness	84 (100)	80 (96)
The program kept my attention	Affective attitude	83 (99)	76 (92)
I learned useful information from the program	Effectiveness	83 (99)	82 (99)
The program covered topics that are important to me	Ethicality	83 (99)	78 (94)
Doing the program was time well spent	Opportunity cost	84 (100)	75 (90)
I understood how to do the program	Self-efficacy	84 (100)	83 (100)
The program took too long	Burden	21 (25)	31 (37)

^a^Items were modified for participants (n=4) who did not complete the first mother-adolescent module of the program so that instead of referring to “the program” the questions referred to “the parts of the program that I did.” Mother participants who did not complete the first mother teen module were not asked item 2.

^b^N=80.

^c^N/A: not applicable.

#### e-MTSD Module Acceptability

The endorsement of acceptability indicators for each of the individual program modules, assessed via surveys that were embedded in the program at the end of each module, was also high (Table 5). Across all program modules between 87% and 100% of mothers and between 81% and 97% of adolescents indicated they agreed that they enjoyed doing the module, the module was easy to do, they learned useful information for the module, and the module kept their attention. Endorsement of the indicator “this module kept my attention” was generally lower among adolescents than among mothers and was lowest for modules 1, 2, and 3. The proportion of adolescents who agreed with the indicator “this module is too long” ranged between 32% (module 4; 23/73) and 47% (module 2; 35/75), indicating lower general levels of acceptability for this indicator, particularly for modules 1, 2, and 3. The percentage of participants who indicated that there were any parts of the program modules that they did not like doing ranged from 4% (Getting Started) to 19% (module 2). We reviewed responses to the open-ended query and flagged specific concerns that were identified by at least 2 respondents who suggested potential revisions to the program content or delivery that could potentially be made to increase program acceptability (Table S4 in Multimedia Appendix 1). Examples of specific potential revisions include reducing the number of video scenarios presented in modules 2 and 3 to address concerns about module length or burden, revisions to increase usability of an activity in module 2 where participants identify conflict resolution skills, and inclusion of additional resources and support to help caregivers and non–lesbian, gay, bisexual, transgender, queer adolescents support youth who identify as lesbian, gay, bisexual, transgender, queer.

**Table 5:** Proportion of mothers and adolescents who agree or strongly agree with indicators of acceptability of each web-based Moms and Teens for Safe Dates program module^a^.

Acceptability indicator (theoretical domain)	Getting Started: mother (n=93), n (%)	Module 1 (n=88), n (%)		Module 2 (n=75), n (%)		Module 3 (n=75), n (%)		Module 4 (n=73), n (%)		Module 5 (n=75), n (%)	
		Mother	Adolescent	Mother	Adolescent	Mother	Adolescent	Mother	Adolescent	Mother	Adolescent
I enjoyed doing this module (affective attitude)	88 (95)	88 (100)	83 (94)	65 (87)	67 (89)	73 (97)	69 (92)	66 (90)	65 (89)	72 (96)	69 (92)
This module was easy to do (burden)	92 (99)	84 (95)	84 (95)	68 (91)	71 (95)	73 (97)	73 (97)	69 (95)	69 (95)	73 (97)	69 (92)
I learned useful information from this module (effectiveness)	91 (98)	87 (99)	83 (94)	71 (95)	68 (91)	72 (96)	71 (95)	72 (99)	68 (93)	72 (96)	72 (96)
This module kept my attention (affective attitude)	89 (96)	88 (100)	74 (84)	70 (93)	61 (81)	73 (97)	63 (84)	71 (97)	66 (90)	72 (96)	69 (92)
This module was too long (burden)	21 (23)	21 (24)	31 (35)	21 (28)	35 (47)	24 (32)	30 (40)	14 (19)	23 (32)	19 (25)	25 (33)
There were parts of this module I disliked doing	4 (4)	10 (11)	13 (15)	14 (19)	13 (17)	5 (7)	7 (9)	6 (8)	7 (10)	8 (11)	5 (7)

^a^The table reports the number (percentage) of participants who reported that they “agree” or “strongly agree” with each statement. The denominators for each module differ depending on the number of dyads that completed the module survey.

#### e-MTSD Program Impacts

Table 6 reports the proportion of mothers and adolescents who endorsed (ie, agreed with) indicators of program impact corresponding to program learning objectives. Over 90% (of mothers and adolescents agreed with each of the program impact indicators except for “the program taught me more about what dating is like for teens today,” which was endorsed by 93% of mothers but only 81% of adolescents. Notably, nearly all mothers and adolescents reported that the program helped them to talk about safe relationships with each other (98% of mothers and 98% of adolescents) and that they would use information from the program in the future (100% of mothers and 98% of adolescents). Furthermore, 100% of adolescents reported that the program taught them “the importance of consent in a dating relationship.”

**Table 6:** Proportion of mothers and teens who completed at least one program module and responded to the postprogram survey (n=80 mothers and n=79 adolescents) and who reported agreement with indicators of impact of the web-based Moms and Teens for Safe Dates program.

Program impact indicators	Mother, n (%)^a^	Adolescent, n (%)^a^
The program taught me how to communicate more effectively with my teen [mom]^b^	75 (94)	73 (92)
The program taught me more about what dating is like for teens today	74 (93)	64 (81)
The program helped me talk about safe dating relationships with my teen [mom]	78 (98)	77 (97)
The program taught me how to manage anger and conflict with others	74 (93)	74 (94)
The program taught me how to recognize dating abuse	77 (96)	78 (99)
The program taught me characteristics of healthy relationships	79 (99)	75 (95)
The program taught me how to [help my teen] avoid becoming involved in dating abuse	78 (98)	77 (97)
The program helped me [my mom and I] to set family guidelines about dating with my teen	74 (93)	72 (91)
The program taught me how to be respectful to the people I date	N/A^c^	77 (97)
The program taught me the importance of consent in a dating relationship	N/A	79 (100)
The program taught me where to seek help if I experienced dating abuse	N/A	73 (92)
I will use information from this program in the future	80 (100)	77 (97)

^a^Of the 88 mother-adolescent dyads who completed at least one module of the program, 80 (91%) mothers and 79 (90%) teenagers completed the postprogram survey. Proportions (%) denote the number of respondents who reported agreeing or strongly agreeing with the indicator relative to the total number of respondents (80 mothers and 79 adolescents).

^b^The text in brackets denote word changes for the mother and adolescent survey questions. These questions were only included in postprogram surveys for respondents who had completed at least 1 of the mother-adolescent program modules.

^c^N/A: not applicable.

#### Acceptability of Adherence Supports

All the participant mothers assigned to the TR and TRAP groups who responded to the postprogram survey and reported receiving SMS text messages (n=39) indicated that the messages were helpful for reminding them to do the program with their adolescent and 92% (36/39) reported that the number of messages received was “just right.” Two mothers reported that the number of SMS text messages was “too many,” and 1 mother reported that the number of messages was “too few.” Of the 50 participants who were assigned to the AP and TRAP conditions, 76% (n=38) completed an action plan. Just over half (n=25, 58%) of the mothers assigned to the AP and TRAP conditions who completed the postprogram survey (n=43) reported that making an action plan was “very helpful” to them in completing the program; 28% (n=12) reported that it was “somewhat helpful”; and 14% (n=6) reported that it was “not very helpful”; no one reported that it was “not at all helpful.”

## Discussion

### Principal Findings

This study examined and found support for the feasibility and acceptability of a web-based family-based dating abuse prevention program for adolescents who have been exposed to IPV. This study has several key findings. First, it is feasible to (1) recruit IPV-exposed adolescent study participants and their maternal caregivers using a multipronged promotion strategy targeted at potentially eligible participant mothers and (2) evaluate program impacts among mothers and adolescents using web-based surveys (research process feasibility). Second, it is feasible for mothers and their adolescents to access and complete the program via a web-based platform (program feasibility). Third, a high proportion of mother and adolescent participants found the program content to be acceptable and agreed that the program impacted targeted learning outcomes (program acceptability). The results also suggest the need to address technical problems identified with the functionality of the program and potential revisions to the program content. We discuss these main findings, including their implications for future e-MTSD program development and evaluation research, in detail in subsequent sections.

### Research Process Feasibility

Findings indicate the practicability of using the recruitment procedures used in this study in future research examining the efficacy of the e-MTSD program. We were able to recruit and fully enroll approximately 18 eligible mother-adolescent dyads per month over a 6-month period, and the enrollment rate was only slightly lower than that achieved in the RCT of MTSD, which enrolled an average of 22 mother-adolescent pairs per month over an 18-month period using a recruitment strategy that relied principally on advertising through domestic violence coalitions and mass transit systems in 2 cities [7]. It is notable that we classified 30 screener respondents, most of whom reported learning about the study via Facebook (n=23), as potentially fraudulent based on the following indicators: multiple screeners with the same contact information, IP address trace to a non-US location, inconsistent or illogical responses to screener questions about the adolescent participant’s age or education level, and email communication with suspicious grammar or syntax duplicated across multiple screener respondents. Our enrollment procedures involved a telephone screen, which likely deterred some fraudulent participation by allowing us to ensure that participants were human (vs bots) and confirm their eligibility. However, a growing body of research using web-based recruitment methods has documented fraudulent participation, suggesting that any future e-MTSD evaluation research that uses web-based recruitment should develop a detailed protocol for identifying potentially fraudulent cases [17,18]. Furthermore, emerging research demonstrates the promise of using “community champions” who are trusted by the target population and have a large web-based following to disseminate study information. For example, a study evaluating a web-based family-based program for Hispanic families found that this strategy was more effective than Facebook advertisements and page promotions for recruitment [19].

The convenience sample enrolled in this study includes some groups that are over- or underrepresented compared with the national data. Specifically, the national data suggest that our study population of mothers was more highly educated than the national average. In 2020, the proportion of people aged ≥25 years in the United States who had at least a bachelor’s degree was 38%, compared with 50% in this study [20]. Furthermore, our study enrolled an adolescent sample that was disproportionately female; approximately 51% of the United States youth aged 10 to 19 years identified as male in 2019, compared with 33% of adolescents in this study [21]. In terms of race and ethnicity, the proportion of adolescents in the United States who identified as non-White in 2019 was 49%, which is similar to the proportion found in this study (53%) [21]. Compared with US Census data, a larger proportion of the adolescent sample in this study identified as non-Hispanic Black (18% vs 14%) and non-Hispanic multiracial (9% vs 4%) and a smaller proportion identified as Hispanic (15% vs 25%) and non-Hispanic White (47% vs 51% [20]). In terms of sexual identity, national data from 2021 found that 74% of US high school adolescents identified as heterosexual, 3% as gay or lesbian, and 12% as bisexual, proportions similar to those found in this study (71%, 4%, and 13%, respectively) [22]. Taken together, these findings suggest that future research to examine the efficacy of the e-MTSD program should take care to ensure that the sample recruited is inclusive of mothers from a range of educational backgrounds, given that maternal education is an indicator of socioeconomic status [23]. Furthermore, recruitment should use strategies (eg, stratified assignment) to ensure adequate participation of male adolescents and to ensure the racial and ethnic diversity of the sample, with particular attention to ensuring adequate representation of Hispanic adolescents. Efforts to ensure the socioeconomic, gender, and racial and ethnic diversity of the sample enrolled to evaluate future iterations of e-MTSD are critical, given that each of these factors has been found to moderate the impacts of violence exposure on trauma symptomology and thus may potentially moderate the impacts of e-MTSD program activities on targeted mediators and the impacts of changes in targeted mediators on violence exposure outcomes [24-27]. We would also like to note that currently e-MTSD is available only in English. Translating the program would potentially increase program reach to families who speak Spanish, although efforts along these lines should carefully consider whether deep-structure adaptations would be needed to ensure the program meets the needs of Spanish-speaking families [28].

Rates of enrollment and retention for this study were in line with or higher than those reported in other studies of web-based family-based programs, suggesting the practicability of our procedures for consent and data collection [29-31]. Notably, however, of the 17 participants who did not complete enrollment after eligibility confirmation, 10 completed consent forms but did not complete a baseline survey. It is unclear whether this can be attributed to baseline survey length; however, it is notable that median baseline survey times (22 min for mothers; 25 min for adolescents) exceed the maximum length for web surveys recommended by market research experts [32,33].

### Program Feasibility

The rates of program completion were higher than those reported for the RCT of the MTSD booklets, supporting the feasibility of web-based delivery of program content. Specifically, 75% of families in this study completed all 6 program modules, whereas only 62% of families completed the last program booklet in the MTSD RCT [7]. The rates of program completion were also higher than those reported in other studies of web-based family-based programs [29,31]. For example, Bourdeau et al [29] tested a 3-module web-based intervention designed to promote parent-adolescent communication about relationships and sexuality in an RCT. Of those assigned to the intervention condition, 14% never logged into the program and 43% completed only 1 to 2 program modules [29]. Median times for completing e-MTSD program modules (16-28 min) are in line with or shorter than those reported in other studies of technology-delivered family-based programs [15,19,29,34]. Notably, however, a significant proportion of adolescents reported that the modules were “too long,” particularly in reference to modules 2 and 3, which took the longest amount of time to complete. Therefore, future iterations of the program should consider whether modules can be shortened or designed to include core and optional components while maintaining fidelity to the underlying program model.

### Program Acceptability

On average, both mothers and adolescents rated the program as highly acceptable across a range of indicators. Nearly all mother participants (98%) reported that the program taught them how to help their teenager avoid dating abuse, a key learning outcome, and 100% of mothers reported that they would use information from the program in the future. When examining ratings across modules, the findings suggested that ratings were slightly lower for module 2 than for the other modules, particularly for indicators tapping into the affective attitude dimension of acceptability. Therefore, future iterations of the program should consider how to make the content of this module more engaging and enjoyable for adolescents. Additional issues to address include technical problems related to program navigation (eg, skipping of the Getting Started module and program not saving progress) and glitches with specific activities.

### Conclusions

Despite a robust field of study of dating violence interventions that use traditional forms of delivery, the field of digital health interventions for the prevention of relationship violence is only just emerging [35]. This study supports the promise of e-MTSD, a 6-module web-based dating violence prevention program for IPV-exposed adolescents and their maternal caregivers. The program is feasible for delivery and acceptable to mothers and adolescents. Future research that includes a demographically diverse population of mothers and adolescents is needed to evaluate the efficacy of the program on targeted outcomes, identify strategies that support program engagement, and determine whether program engagement and outcome impacts vary among mothers and adolescents with different sociodemographic characteristics and family experiences.

## Appendix

Multimedia Appendix 1 Items assessing participant characteristics and technological problems identified with the web-based program.

## Abbreviations

AP: action planning only
CONSORT: Consolidated Standards of Reporting Trials
e-MTSD: web-based Moms and Teens for Safe Dates
IPV: intimate partner violence
LA: low adherence support
MTSD: Moms and Teens for Safe Dates
RCT: randomized controlled trial
TR: text reminders only
TRAP: text reminders plus action planning

## Data Availability

The data sets analyzed as part of this study are available from the corresponding author upon reasonable request.
