# Supplementary material for: Web-Based Delivery of a Family-Based Dating Abuse Prevention Program for Adolescents Exposed to Interparental Violence: Feasibility and Acceptability Study
Source: JMIR Form Res. 2023 Dec 1;7:e49718. doi: 10.2196/49718 (PMC10724814; doi:10.2196/49718)
Supplement: Multimedia Appendix 1 [file formative_v7i1e49718_app1.docx]

**Online Appendix**

Table 1. Questions, sources, and response options for indicators assessing mother participant characteristics

| **Characteristic** | **Question(s) [source if from an established questionnaire or scale]** | **Response Options** |
| --- | --- | --- |
| Relationship to teen | What is your relationship to this teen? | 1. Biological /adoptive mother 2. Stepmother 3. Grandmother 4. Other |
| Age in years | How old are you? (in years) | Numerical entry |
| Ethnicity | Do you consider yourself to be Hispanic or Latino/a? | 1. Yes 2. No |
| Race | How would you describe yourself? | 1. American Indian or Alaska Native 2. Asian 3. Black and African American 4. Native Hawaiian or Other Pacific Islander 5. White or Caucasian 6. Multi-racial 7. Other |
| Educational attainment | What is the highest degree or level of school you have completed? | 1. No schooling completed 2. Nursery School 3. Grades 1 through 11 4. 12^th^ grade – no diploma 5. Regular High School Diploma 6. GED or alternative credential 7. Some college credit, but less than 1 year of college 8. 1 or more years of college credit, no degree 9. Associates degree (for example: AA, AS) 10. Bachelor’s degree (for example: BA, BS) 11. Master’s Degree (for example: MA, MS, MEng, MEd, MSW, MBA) 12. Professional degree beyond bachelor’s degree (for example MD, DDS, DVM, LLB, JD) 13. Doctorate degree (for example, PhD, EdD) |
| Currently living with a partner | Do you have a partner who currently lives with you? | 1. Yes 2. No |
| Somewhat or very worried about family having enough to eat in the next month | Thinking about the next month, how worried are you that you and your family will have difficulty with the following: Having enough to eat [12] | 1. Very worried 2. Somewhat worried 3. Not too worried 4. Not at all worried |
| Ever filed for a domestic violence protective order | Did you ever file for a domestic violence prevention order against this partner? | 1. Yes 2. No |
| Ever pushed, slapped, hit, punched, kicked, choked, or beat up by a partner | At any time in your life did a partner ever push, slap, hit, punch, kick, choke, or beat you up? | 1. Yes 2. No |
| Ever repeatedly sworn at, insulted, or humiliated by a partner | At any time in your life did a partner ever repeatedly swear at, insult, put down, or humiliate you? | 1. Yes 2. No |
| Ever forced to do sexual things they did not want to by a partner | At any time in your life did a partner ever force you to do sexual things you did not want to? | 1. Yes 2. No |

Table 2. Questions, sources, and response options for indicators assessing adolescent participant characteristics

| **Characteristic** | **Question(s) [source if from an established questionnaire or scale]** | **Response Options** |
| --- | --- | --- |
| Age in years | How old are you? (in years) | Numerical Entry |
| Gender identity | How would you currently describe yourself?  [13] | 1. Male 2. Female 3. Transgender 4. Genderqueer/ Gender non-conforming 5. Different Gender Identity |
| Race | How would you describe yourself? | 1. American Indian or Alaska Native 2. Asian 3. Black and African American 4. Native Hawaiian or Other Pacific Islander 5. White or Caucasian 6. Multi-racial 7. Other |
| Ethnicity | Do you consider yourself to be Hispanic or Latino/a? | 1. Yes 2. No |
| Sexual identity | How would you describe your sexual identity? [13] | 1. Gay or lesbian 2. Bisexual 3. Straight or heterosexual 4. Something else |
| Adolescent IPV exposure | Ever seen or heard mother get pushed, slapped, hit, punched, kicked, choked, or beat up by a partner  Mother answered “yes” to both questions:  *At anytime in your life did a partner ever push, slap, hit, punch, kick, choke, or beat you up?*  *At anytime in their life, did your teen ever see or hear this happen to you?* | 1. Yes 2. No |
| Adolescent IPV exposure | Ever seen or heard mother be repeatedly sworn at, insulted, or humiliated by a partner  Mother answered “yes” to both questions:  *At anytime in your life did a partner ever repeatedly swear at, insult, put down, or humiliate you?*  *At anytime in their life, did your teen ever see or hear this happen to you?* | 1. Yes 2. No |
| Adolescent dating status | Ever dated, gone out, hooked up, or been in a romantic relationship with someone  Answered yes to the following question:  *In your whole life, have you ever dated, gone out, hooked up, or been in a romantic relationship with someone? This could be a boyfriend or girlfriend or someone you were talking to or hanging out with even if it was not serious* [14] | 1. Yes 2. No |
| Psychological dating violence victimization^a^ | Answered yes to any of the below questions:   - *They insulted my looks, clothes, or appearance, and it made me feel bad, embarrassed, or insecure [14]* - *They threatened to hurt me, which scared or worried me [7]* ^b^ - *They hurt my feelings on purpose [7]* - *They spread rumors, gossip, or secrets about me using texts, social media or apps [14]* - *They called me names, put me down, or said really mean things to me using texts, social media or other apps [7]* | 1. Yes 2. No |
| Physical dating violence victimization^a^ | Answered yes to any of the below questions:   - *They twisted my arm, slapped, pushed, shoved, or shook me [14]* ^b^ - *They hit, punched, kicked or choked me [14]* - *They used a stick, bat, knife, gun or other weapon on me [14]* ^b^ | 1. Yes 2. No |
| Sexual dating violence victimization^a^ | Answered yes to any of the below questions:   - *They pressured me to do something sexual [14]* - *They asked or pressured me for a nude or almost nude photo or video of me, when I did not want to give them one [14]* - *They showed or sent other people nude, or almost nude, photos or videos of me and I did not want them to do that [14]* - *They forced me to do something sexual [14]* - *They gave me alcohol or drugs in order to get sexual with me when I did not want to get sexual [14]* | 1. Yes 2. No |
| Psychological dating violence perpetration^a^ | Answered yes to any of the below questions:   - *I insulted their looks, clothes, or appearance, to make them feel bad, embarrassed, or insecure [14]* - *I threatened to hurt them to scare or worry them [7]* ^b^ - *I hurt their feelings on purpose [7]* - *I spread rumors, gossip, or secrets about them using texts, social media or apps [14]* - *I called them names, put them down, or said really mean things to them using texts, social media or other apps [7]* | 1. Yes 2. No |
| Physical dating violence perpetration^a^ | Answered yes to any of the below questions:   - *I twisted their arm, slapped, pushed, shoved, or shook them [14]* ^b^ - *I hit, punched, kicked or choked them [14]* - *I used a stick, bat, knife, gun or other weapon on them [14]* ^b^ | 1. Yes 2. No |
| Sexual dating violence perpetration^a^ | Answered yes to any of the below questions   - *I pressured them to do something sexual [4]* - *I asked or pressured them for a nude or almost nude photo or video of themselves, when they might not have wanted to give me one [14* - *I showed or sent other people nude or almost nude photos or videos of them that they didn’t know about or might not have wanted me to do [14]* - *I forced them to do something sexual [14]* - *I gave them alcohol or drugs in order to get sexual with them because they might not have wanted to get sexual [14]* | 1. Yes 2. No |

^a^For dating violence questions adolescents were asked “think about all of the people you have ever dated, gone out with, hooked up, or been in a romantic relationship with. These people could include boyfriends or girlfriends or people you were talking to or hanging out with even if it was not serious. Answer the following questions thinking about these people. Remember your responses are confidential and will not be linked to your personal identity. In your whole life, did any of the following things happen?”

^b^Original item wording modified for current study.

Table 3. Technical Problems with eMTSD Identified by Participants in Module Completion Surveys

| Technological Problems Identified [specific module(s) where this concern arose] |
| --- |
| - Couldn’t get sound on videos or narrated portions and/or sound not loud enough [Getting Started, Module 2, Module 3, Module 4] |
| - After page refresh or log out participant made to start over [Getting Started, Module 1] |
| - Skipped Getting Started [Getting Started] |
| - Activity glitch when making selections [Module 1] |
| - After rating an activity made to start over [Throughout Program] |
| - Activity glitch when answering questions [Module 2] |
| - Activity glitch when completing puzzle [Module 3] |
| - Section incorrectly labeled [Module 4] |

Table 4. Participant Concerns Related to Module Content and Potential Revisions to Increase Program Acceptability

| Program Module: Title | Content related issue or concern  (no. of participants who identified this issue/concern) | Potential Revision |
| --- | --- | --- |
| Getting Started | - Actor readings of testimonials did not sound authentic (2 moms). | - Review and revise narratives and re-record with review by mother and adolescent advisors to rate perceptions of authenticity. Possibly include video testimonial(s) |
| Module 1: Talking about Dating | - Module was long and a lot to sift through (2 moms) - Module involved a lot of talking (2 teens) - Module mother-adolescent communication helpers and blockers video scripts did not seem realistic (2 teens) | - Shorten module to include fewer video scenarios. - Review video scripts with adolescent advisors and potentially revise to increase authenticity and salience |
| Module 2: Skills for Handling Conflict | - Activity where participants were asked to identify conflict resolution skills being enacted was confusing and difficult to complete (5 moms; 2 teens) - Conflict resolution scenarios may be reflective of red flag behavior that may escalate into abuse (2 moms; 1 teen) - Content not engaging (3 teens) | - Revise the conflict resolution skills identification activity instructions and content to be more straightforward - Review and revise introductory video and conflict resolution activity scenarios together with mother advisors to include content on red flag behavior and boundary setting in relationships - Review module with adolescent advisors to identify ways to potentially deliver content in a more engaging way and increase relevance to teens |
| Module 3: Recognizing Dating Abuse | - Because of biblical views, discomfort with portrayal of LGBTQ relationships (2 moms) | - Include content in Getting Started stating that LGBTQ relationships will be portrayed in the program because: (1) adolescents in the US express and experience great diversity in gender and sexual identities and expression and (2) all adolescents, including those who are diverse in their sexuality and gender identities and expression, deserve safe and healthy dating relationships - Provide more tailored information and resources to caregivers to help them support LGBTQ+ youth in their or their teen’s lives |
| Module 5: Planning for the Future | - Last section reviewing program content was too repetitive (2 moms, 1 teen) | - Make the program review section optional for those who want to review what they learned |
